# Outbreaks of *Elizabethkingia miricola* Caused Fatal Meningitis-Like Disease in Cultured Bullfrogs

**DOI:** 10.1155/2024/4733320

**Published:** 2024-04-22

**Authors:** Rui An, Guanxin Hou, Xinyi Sun, Lili Wang, Chunxiao Zhang, Yajing Han, Yonghui Li, Tonglei Wu, Qiumei Shi, Zhigang Zhu, Zhiqiang Zhang

**Affiliations:** ^1^Hebei Key Laboratory of Preventive Veterinary Medicine, Hebei Normal University of Science and Technology, Qinhuangdao, Hebei, China; ^2^The Second Hospital of Qinhuangdao, Qinhuangdao, Hebei, China; ^3^Tianjin Dabeinongchangnong Aquatic Technology Co., Ltd., Tianjin, China

## Abstract

*Elizabethkingia miricola* is an emerging nosocomial pathogen responsible for meningitis, sepsis, urinary tract infection, pneumonia, and joint infection in humans. These pathogens were also reported to be causal agents for meningitis-like disease in cultured frogs, which displayed high infectivity, mortality, and significant loss. In July 2023, 10 outbreaks of infectious meningitis-like disease in bullfrogs occurred in Tangshan area. To determine the causal agent, 70 diseased frogs from 10 farms were collected for etiological identification. Gram-negative bacilli were isolated from the brain and liver of sick bullfrogs and identified as members of *E. miricola* by biochemical characterization and 16S rRNA sequencing analysis. A total of 42 strains of *E. miricola* were isolated and further determined as the etiological agent by reproducing neurological symptoms and deaths in an artificial infection test. A representative isolate, HBTS-1, was picked up for the pathogenicity test, and the data showed that this stain was highly pathogenic to bullfrogs with an LD_50_ of 3.7 × 10^5^ CFU. Notably, the isolate also showed high pathogenicity to 5-day-old suckling mice, with an LD_50_ of 3.1 × 10^6^ CFU, indicating its potential threat to mammals. Moreover, all the 42 *E. miricola* isolates showed resistance to multiple antibotics without an apparent inhibition zone observed in the test, making the choice of antimicrobial therapy challenging. These novel findings prioritized *E. miricola* as an important zoonotic agent, which may provide a reference for human medicine.

## 1. Introduction

Bullfrog, the colloquial name for *Rana catesbeiana*, is a native specie of eastern North America and has been widely introduced around the world as a high-quality protein source [1]. Owing to the strong market demand, outstanding reproductive performance, and fast growth rate, the bullfrog aquaculture developed rapidly, and the bullfrog has become the most farmed amphibian globally [2]. However, due to the increased breeding density and poor water environment, increased outbreaks of emerging infectious diseases have occurred on farmed bullfrogs, causing significant losses and posing a major threat to the bullfrog industry [3].

Crooked head disease is an emerging communicable neurologic disease in multifrog species, including black-spotted frog *Pelophylax nigromaculatus* [1], spiny frog *Quasipaa spinosa* [2], Chapa bug-eyed frog *Theloderma bicolor*, northern leopard frog *Lithobates pipiens*, dwarf frogs *Hymenochirus curtipes* [3], warty toad *Bombina microdeladigitora*, and Sabana Surinam toad *Pipa parva* [4]. The suffered frogs present similarly meningitis-like manifestations, including anorexia, crooked head, bent body, torticollis, swimming in circles, and lethargy, and some individuals display cataracts, meteorism, and skin hyperemia [4]. Most diseased frogs would die within several days to weeks after the onset of illness [1, 2]. This emerging disease, with high infectivity and mortality, has brought great threats and losses to the frog industry. Some species of the genus *Elizabethkingia* mainly *Elizabethkingia miricola* and *Elizabethkingia meningoseptica* are reported responsible for crooked head disease in frogs [5].

*E. miricola* is Gram-negative, aerobic, pale yellow-pigmented, nonmotile, non-spore-forming, oxidase-positive, and nitrate-negative bacilli and was ubiquitously distributed in natural environments such as water, soils, fish, frogs, and insects [6, 7]. As a medically important species of *Elizabethkingia*, this bacterium was reported to cause multitype infections in humans, especially in immunocompromised populations and newborn children, such as encephalitis [8], sepsis [9], bacteremia [10], joint infection [11], urinary tract infection [12], and pulmonary abscess [3]. Recently, the reports about *E. miricola*-caused infection in other animals, such as dogs, cats, and frogs [13], are increasing, indicating this bacterium may be an important zoonosis pathogen. However, the literature about cross-species infection of *E. miricola* is rare. Here, we reported outbreaks of crooked head disease in cultured bullfrogs in Tangshan city, Hebei Province, China. From the sick frogs, we isolated strains of *E. miricola* and demonstrated their high pathogenicity to both bullfrogs and suckling mice.

## 2. Materials and Methods

### 2.1. Epidemiological Survey of Crooked Head Disease in Bullfrogs on Farms

Clinical symptoms of crooked head disease were recorded based on gross lesions and necropsy of bullfrogs from 10 traditional bullfrog farms in Hebei Province. Pathological examination and isolation of pathogenic bacteria were performed after euthanasia of diseased frogs. All procedures were approved by the Animal Protection and Utilization Committee of Hebei Science and Technology Normal University.

### 2.2. Etiological Isolation and Inoculum Preparation

Under aseptic conditions, brain, liver, spleen, eye, blood, and muscle tissue samples of infected bullfrogs were collected and inoculated into LB, Trypticase Soy Agar Medium (TSA) (Hope Biology Co., Ltd, Qingdao) and blood agar media (Kont Biology and Technology Co., Ltd, Wenzhou) and then cultured at 30°C for 24–30 hr. The isolates were purified and then replicated on the TSB liquid medium. Log-phase growing cultures were washed twice with PBS and stored at −70°C until use.

### 2.3. Identification of Clinical Isolates

#### 2.3.1. Physiological and Biochemical Characterization of the Isolates

The isolates were streaked on LB plates and TSB plates, respectively, and cultured at 30°C for 24 hr to determine the growth characteristics and colony morphology. The isolates were incubated at 30 and 37°C, respectively, to determine the effect of incubation temperature on bacterial growth. The hemolytic capacity of the isolates was analyzed by observing the hemolysis on blood agar. Gram's staining was performed to determine the bacterial morphology characteristics. Bacterial microbial biochemical identification tubes (Landbridge Technology Co., Ltd., Beijing) were used to detect the presence of oxidative, lysine decarboxylase, and other biochemical indices in the clinical isolates, and the data were compared with the results of *E. miricola* CM160701 [2].

#### 2.3.2. Molecular Identification of *E. miricola* by 16S rRNA Sequencing

DNA was extracted via commercial DNA Extraction Kit per the manufacturer's instruction (TianGen Biotech (Beijing) Co., Ltd., Beijing). PCR was performed as previously described [14], using primers of 16S rRNA (27F:5′-AGAGTTTGATCCTGGCTCAG-3′; 1492R: 5′-TACGGCTACCTTGTTACGACTT-3′). The reaction was performed under the following conditions: initial denaturation at 94°C for 5 min, followed by 35 cycles of thermal denaturation at 94°C for 30 s; annealing at 55°C for 30 s; and extension at 72°C for 45 s. The amplification products were detected and analyzed by 1.5% agarose gel electrophoresis. The PCR amplification products were sent to Shengong Bioengineering (Shanghai) Co., Ltd., for sequencing, and the DNA sequence data were analyzed using BLAST software (http://blast.ncbi.nlm.nih.gov/Blast.cgi). Phylogenetic analysis was performed based on 16S rRNA genes, and the phylogenetic position for the isolated strain was determined by comparison with related taxa (five *E. miricola* isolates, two *Elizabethkingia anopheles* isolates, two *E. meningoseptica* isolates, and two *Chryseobacteria* strains) obtained from the GenBank database (http://www.ncbi.nlm.nih.gov/). A phylogenetic tree was constructed using the neighbor-joining method in MEGA11 [15]. The topologies of the phylogenetic trees were assessed by bootstrap analysis of 1,000 replications. All ambiguous positions were removed for each sequence pair (pairwise deletion option). There were a total of 1,388 positions in the final dataset [16, 17].

### 2.4. Virulence Gene Testing

PCR amplification of the causative agent was performed to verify the presence of genes coding for virulence components including polar flagella (*fabG*), T4SS effectors (*fabV*), capsule (*wecB*), urease (*ureB*), isocitrate lyase (*aceA*), acyl carrier protein (*acyl*), hemolysin (*hly*), and O-antigen biosynthesis enzyme (*wbpO*), with the specific primers that are listed in Table 1, referring to a previous study [18]. PCR products were analyzed by agarose gel electrophoresis with a 1% agarose gel containing nucleic acid dye (Saibai Sheng Gene Technology Co., Ltd., Beijing).

### 2.5. Bullfrog Infection Test

A total of 96 healthy bullfrogs in eight groups were raised temporarily before the experiment and identified as not carrying *E. miricola* through oral, skin, and anal bacteria identification via PCR with primers [4]. The isolate was inoculated in the TSB medium and incubated at 30°C in a shaker for 12 hr. A 1 mL bacterial sample was taken, the medium was discarded, and then suspended with sterile PBS for bacterial drop plate counting. Each group of bullfrogs was half male and half female for the next test. The bullfrogs in four groups were intramuscularly injected with 0.2 mL of *E. miricola* isolate solution at the doses of 2 × 10^7^ CFU, 2 × 10^6^ CFU, 2 × 10^5^ CFU, and 2 × 10^4^ CFU, respectively. The bullfrogs in the other three groups were injected with 2 × 10^7^ CFU isolates in 0.2 mL sterile PBS by oral, intraperitoneal injection and immersion infection, respectively. The control group was injected with an equal volume of PBS, and the infected bullfrogs were observed for 14 consecutive days. The morbidity, mortality, and pathologic changes of experimental bullfrogs were recorded, and the liver and brain of infected bullfrogs were taken to detect the presence of the isolates.

Five diseased bullfrogs postchallenge with apparent neurologic symptoms, cataracts, and skin erythema were randomly picked up for histopathologic examination. The brain, liver, spleen, kidney, and eye tissues (0.5 cm × 0.5 cm × 0.2 cm) were taken and fixed in 4% paraformaldehyde fixative (Lanjieke Science and Technology Co., Ltd., Beijing) for 48 hr. Routine paraffin sections and HE (hematoxylin and eosin) staining were carried out, and the histopathological changes were observed under the microscope.

### 2.6. Suckling Mouse Infection Test

Thirty 5-day-old KM suckling mice were divided into five groups with six suckling mice per group. Suckling mice of four groups were intramuscularly injected with 0.02 mL isolated bacterial solution at the doses of 2 × 10^8^ CFU, 2 × 10^7^ CFU, 2 × 10^6^ CFU, and 2 × 10^5^ CFU, respectively, and the control group was injected with an equal volume of PBS, 120 hr of continuous observation. The liver and brain of infected mice were homogenized, and the presence of bacteria was detected by PCR [4].

### 2.7. Antibiotic Sensitivity Test

There are currently no established standards for *Elizabethkingia*, and susceptibilities are largely reported based on the Enterobacteriaceae standard of the Clinical and Laboratory Standards Institute (CLSI) M100 guidelines and/or the European Committee on Antimicrobial Susceptibility Testing (EUCAST) pharmacokinetic–pharmacodynamic (PK–PD) [14, 19]. Here, we evaluated the antibiotic susceptibility of *E. miricola* isolate bacteria by referring to the Enterobacteriaceae standard in CLSI M100 guidelines. A total of 22 antibiotics, including ampicillin, cefuroxime, ceftriaxone, ceftazidime, cefaclor, aztreonam, meropenem, streptomycin, gentamicin, kanamycin, amikacin, tetracycline, doxycycline, minocycline, chloramphenicol, florfenicol, trimethoprim, rifampicin, ciprofloxacin, ofloxacin, norfloxacin, and nitrofurantoin, were selected for testing bacterial antibiotic susceptibility. According to the method recommended by CLSI, the Kirby–Bauer method was used to determine the drug resistance spectrum of the isolated strains. Briefly, the isolated bacteria were inoculated in MHB (cation-adjusted Mueller–Hinton broth) and incubated at 30°C on a shaker for 12 hr. Log-phase bacterial cultures were diluted to a 0.5 McFarland unit with normal saline and spread on the surface of MHA (cation-adjusted Mueller–Hinton agar). The standard antibiotic disks (Hangzhou Microbial Reagent Co., Ltd., China) were placed on the surface of the MHA and cultured in a constant temperature incubator at 30°C for 24 hr. The diameter of the inhibition zone of each strain was measured, and the isolates were categorized based on the presence or absence of an inhibition ring.

## 3. Results

### 3.1. Outbreaks of Crooked Head Disease in Farmed Bullfrogs in Tangshan City

**Figure 1:**
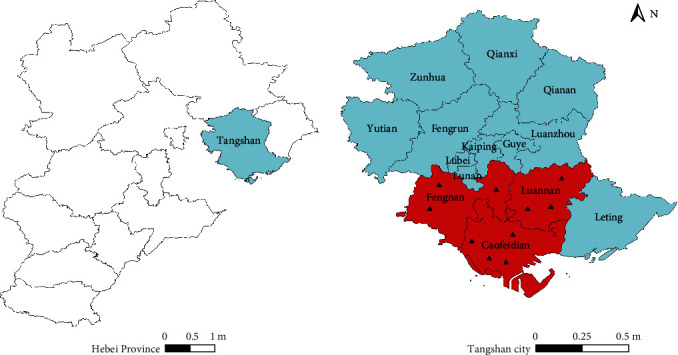
Geographical locations of bullfrog sample collection sites. The blue areas represent Tangshan city, and the red areas represent the sampling region. A total of 42 *E. miricola* isolates were isolated from bullfrog farms in Tangshan, Hebei Province, China, including 12 isolates from 3 farms in Fengnan, 13 isolates from 3 farms in Luannan, and 17 isolates from 4 farms in Caofeidian.

In 2023, an extensive outbreak of crooked head disease occurred in cultured bullfrogs in Tangshan city of Hebei Province, which caused large-scale deaths of bullfrogs and significant loss. To determine the primary causative agent of these diseases, a total of 70 sick frogs from 10 bullfrog farms (Figure 1) of Tangshan region were performed for etiological examination. The information of geographical distribution and coordinates of bullfrog farms sampled is illustrated in *Supplementary 1*.

**Figure 2:**
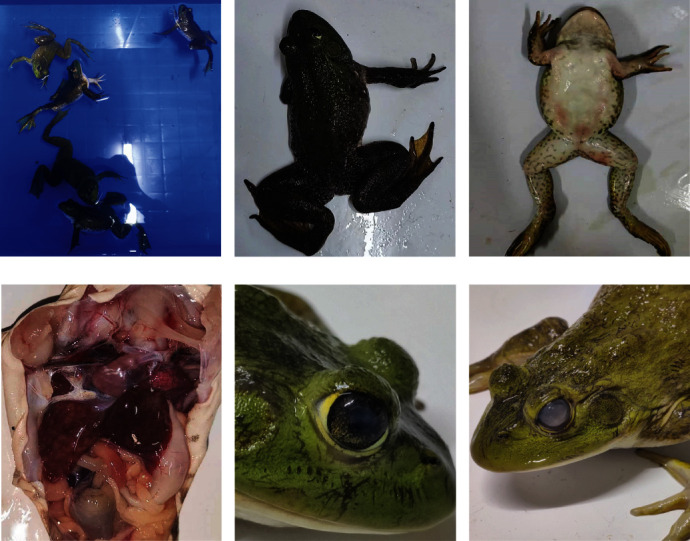
Clinical signs and pathological changes of the sick bullfrogs. (a) The affected frog has an abnormal posture. (b) The head of the sick frog appears to be severely tilted to one side. (c) The infected frog has red bleeding spots on the inner thigh. (d) The livers of the diseased frogs were found to be congested and enlarged with yellow and red spots on the surface. (e and f). The eyes of the sick frog are cloudy and white.

The diseased frogs showed crooked heads, stiff bodies, and dyskinesia (Figures 2(a) and 2(b)), reddish patches on the abdomen and legs (Figure 2(c)), the livers of the diseased frogs were found to be congested and enlarged with yellow and red spots on the surface (Figure 2(d)), and some with turbid and cloudy eyeballs of different degrees (Figures 2(e) and 2(f)).

### 3.2. Strains of *E. miricola* Were Isolated from Brain Tissues of Sick Bullfrogs

The brain tissues of the diseased bullfrogs were collected for bacterial isolation and identification. A dominant strain was isolated and named HBTS-1. This isolate, when challenged by intramuscular injection, was able to cause typical meningitis-like signs in bullfrogs. Koch's postulates were satisfied by isolation of bacteria from dead frogs identical to HBTS-1. Furthermore, we also performed other routes of exposure, such as oral, intraperitoneal injection and immersion, and all these ways of infection could lead to meningitis-like illness and death of bullfrogs, with existence of *E. miricola* in the liver, spleen, and brain of challenged frogs. All these results strongly demonstrated that the strain HBTS-1 was the causative agent of crooked head disease.

**Figure 3:**
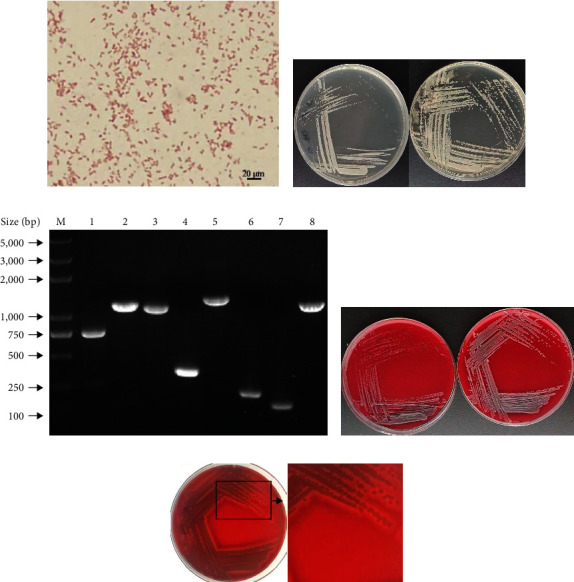
Gram staining, culture, and virulence factor detection of isolated bacteria. (a) Gram stain. (b) Growth in the LB medium at 30°C for 24 hr (left); growth in the TSA medium at 30°C for 24 hr (right). (c) Virulence factor detection (M: DM2000 plus; 1: *fabG*; 2: *fabV*; 3: *wecB*; 4: *ureB*; 5: *aceA*; 6: *acyl*; 7: *Hly*; 8: *wbpO*). (d) On the blood medium, cultured at 37°C for 24 hr (left); the same medium was cultured at 30°C for 24 hr (right). (e) Production of the hemolytic ring in isolated bacteria.

**Figure 4:**
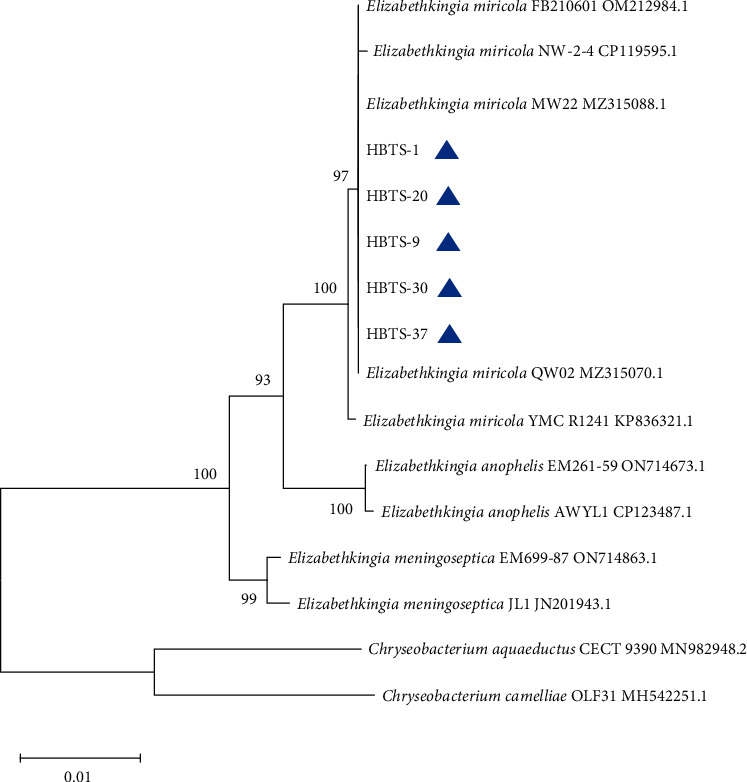
Phylogenomic tree based on 16S rRNA gene sequences.

Then, we performed the identification of the HBTS-1 isolate through morphological and biochemical examination. The isolate showed Gram-stain-negative bacilli under the microscope (Figure 3(a)) and was able to grow on LB, TSA, and blood agar and possessed hemolytic properties (Figure 3(e)). Interestingly, the bacterium was able to grow at 37°C, with a slight retardation as compared to that at 30°C (Figures 3(b) and 3(d)). The data of biochemical examination showed that the biochemical characteristics of the HBTS-1 isolate were similar to those of *E. miricola* [2] (Table 2). To verify the results of biochemical examination, we further analyzed the strain HBTS-1 at a molecular level using the 16S rRNA sequencing assay and also examined the virulence genes of the bacteria. According to 16S rRNA gene sequence similarity and phylogenetic analysis, all the tested isolates including HBTS-1 were classified as members of *E. miricola* (Figure 4). As shown in Figure 3(c), the HBTS-1 strain carried the eight virulence factors of *E. miricola* previously reported. Taken together, these results indicated that the isolate HBTS-1, the causative agent of crooked head disease, was a strain of *E. miricola*.

After that, we examined the prevalence of in sick bullfrogs in Tangshan region. A total of 42 strains of *E. miricola* were isolated from 70 sick bullfrogs of 10 farms, with an isolation rate of 60% (42/70). This result suggested that *E. miricola* is the major cause of crooked head disease of bullfrogs in Tangshan region.

The positions of the five isolates (marked with blue triangles) and reference strains of genus *Elizabethkingia* and *Chryseobacteria* were shown in the phylogenomic tree, and bootstrap values (the percentage of 1,000 data resamplings) ≥50% were shown at the nodes based on the neighbor-joining method. The tree is drawn to scale, with branch lengths in the same units as those of the evolutionary distances used to infer the phylogenetic tree. The evolutionary distances were computed using the Kimura 2-parameter method and are in the units of the number of base substitutions per site.

### 3.3. *E. miricola* HBTS-1 Was Highly Pathogenic to Bullfrogs

**Figure 5:**
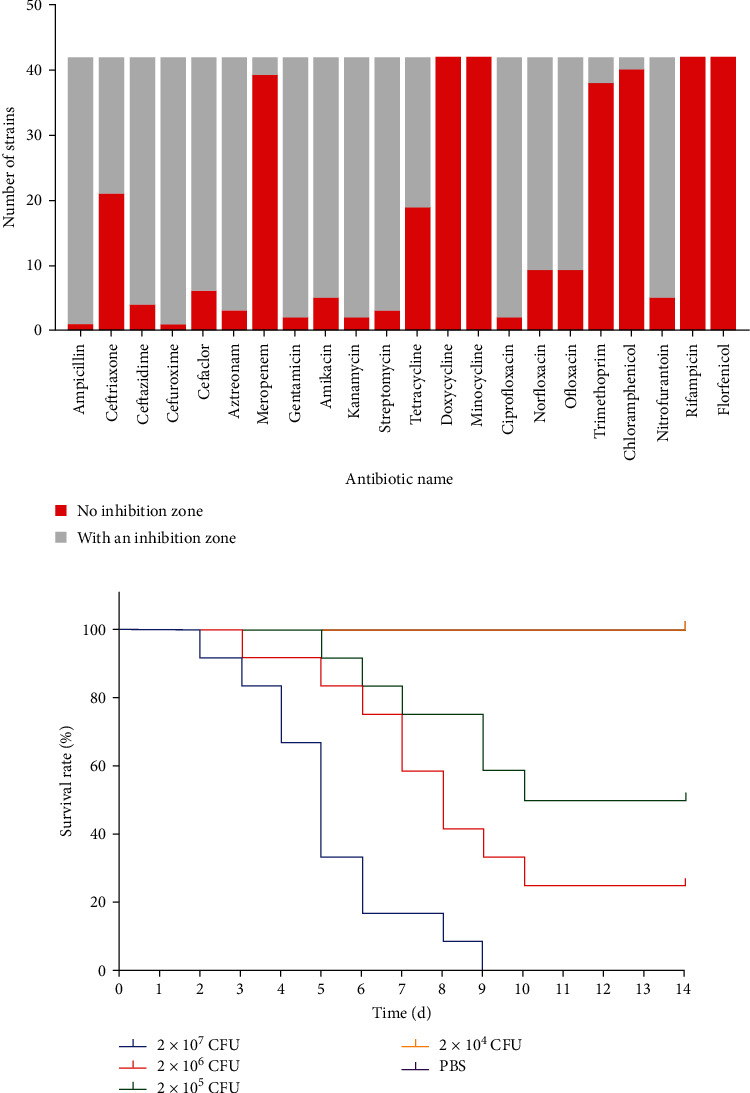
Antibiotic sensitivity and pathogenicity testing for the isolates. (a) The results of antibiotic sensitivity testing of 42 isolates. The isolates were categorized based on the presence or absence of an inhibition ring, and the number of the isolates was recorded. (b) The survival rate of bullfrogs postinfection with *E. miricola* HBTS-1. Bullfrogs were infected with *E. miricola* HBTS-1 at different doses or PBS as controls as described in Section 2.

In order to determine the pathogenicity of *E. miricola* isolates, the HBTS-1 isolate was taken as a representative strain for the bullfrog infection test. When intramuscularly injected with HBTS-1 at the doses of 2 × 10^7^ CFU, 2 × 10^6^ CFU, 2 × 10^5^ CFU, and 2 × 10^4^ CFU, the frogs showed head tilt to one side, mental retarding, cataract, loss of orientation, and even death. The cumulative mortality rate of cultured bullfrogs during the experiment was 100%, 75%, 50%, and 0%, respectively, while bullfrogs of the control group did not get sick and die during the whole experimental observation period (Figure 5(b)). The LD_50_ of HBTS-1 in bullfrogs was calculated as 3.7 × 10^5^ CFU using Karber's method. These results demonstrated that *E. miricola* HBTS-1 was highly pathogenic to the cultured bullfrogs.

**Figure 6:**
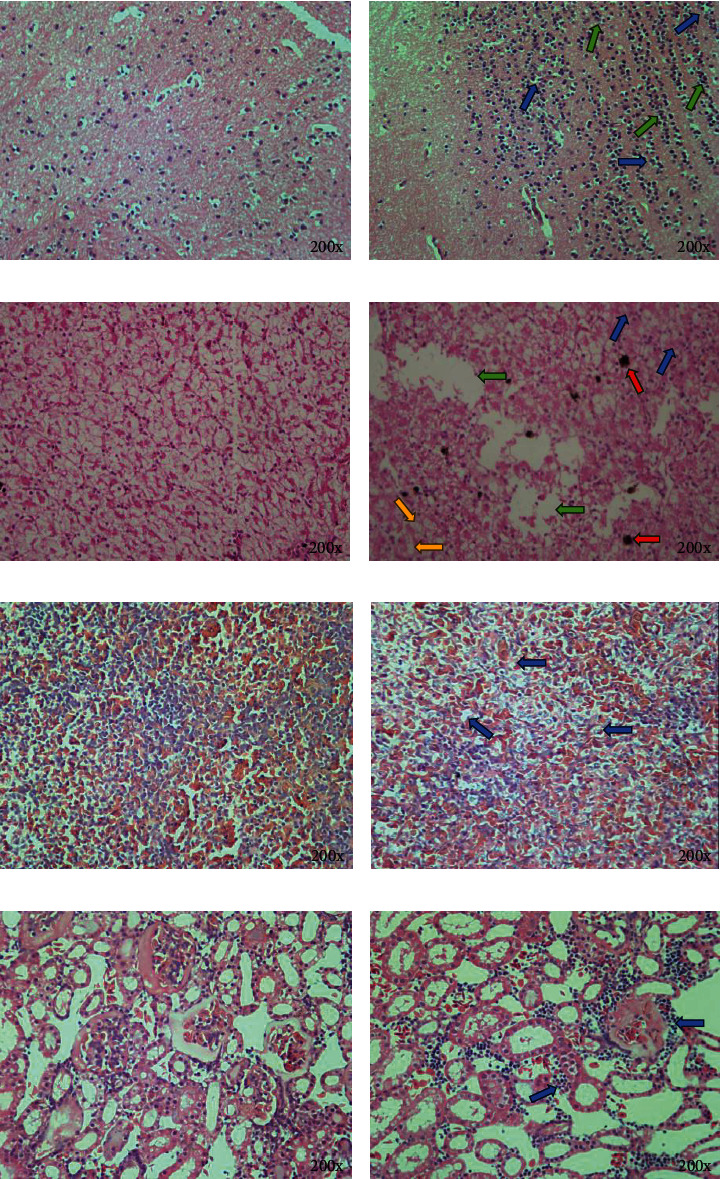
Histopathological analysis of *E. miricola*-infected bullfrogs. (a) Brain tissue of healthy bullfrogs. (b) Brain tissue of a diseased bullfrog: increased glial cells (green arrow) and increased vacuoles in the interstitium (blue arrow). (c) A healthy bullfrog's liver. (d) Liver of diseased bullfrog: blurring of the cytoplasmic structure of hepatocytes (blue arrows), the appearance of varying numbers of vacuoles (yellow arrows), disappearance of collapsed hepatocytes (green arrows), and a large amount of brown pigmentation (red arrows). (e) Spleen of a healthy bullfrog. (f) Spleen of diseased bullfrog: spleen showing vacuolar degeneration (blue arrow). (g) Kidneys of healthy bullfrogs. (h) Diseased bullfrog kidneys: the kidneys are infiltrated with a large number of inflammatory cells.

Tissue samples from dying bullfrogs were collected for pathological analysis. The results revealed that the brain glial cells of the affected bullfrogs were increased and there were vacuoles in the stroma (Figures 6(a) and 6(b)); the cytoplasmic structure of the affected frogs' hepatocytes was blurred, there were a varying number of vacuoles, the hepatocytes collapsed and disappeared, and there were a lot of brown pigmentation (Figures 6(c) and 6(d)); the spleens of the frogs had vacuolar degeneration (Figures 6(e) and 6(f)); and the kidneys had a large number of inflammatory cell infiltration (Figures 6(g) and 6(h)).These results indicated that the *E. miricola* isolate could cause multiorgans injures in bullfrogs.

### 3.4. *E. miricola* HBTS-1 Displayed Pathogenicity to Suckling Mouse

**Figure 7:**
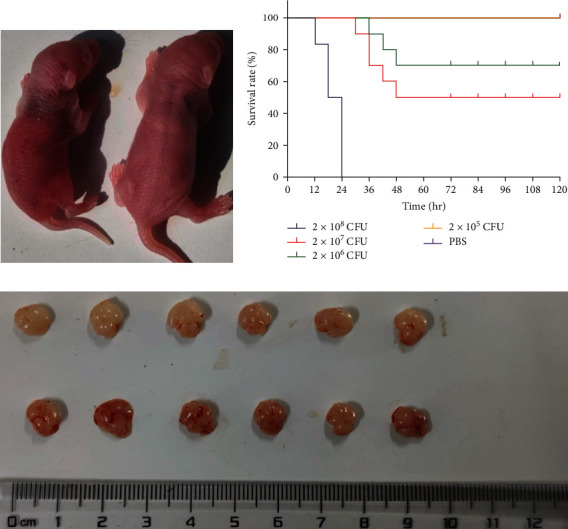
Study on suckling mice inoculated with clinical isolate *E. miricola* HBTS-1. (a) Infected suckling mice (left) and control mice (right). (b) Kaplan–Meier survival curves of suckling mice infected with different doses of HBTS-1. Control group/phosphate-buffered-saline (PBS), the concentrations in the infection group were 2 × 10^5^ CFU, 2 × 10^6^ CFU, 2 × 10^7^ CFU, and 2 × 10^8^ CFU of HBTS-1. (c) Brains of infected suckling mice (bottom 6) and a control group of suckling mice (top 6).

As the HBTS-1 isolate has the ability to grow at 37°C, the body temperature of mammals, we also determined its pathogenicity in mice. We infected adult KM mice and 5-day-old suckling mice with the HBTS-1 isolate by intramuscular injection. The data showed that the HBTS-1 isolate failed to establish an infection in adult mice without any abnormal performance and existence of bacteria in organs. While the suckling mice, when received inoculation of the HBTS-1 isolate, developed red bodies and cerebral hemorrhage and eventual death (Figures 7(a) and 7(c)), with existence of *E. miricola* detected in livers and brain tissues. As shown in Figure 7(b), all the suckling mice (6/6) received intramuscular injection with a dose of 2 × 10^8^ CFU died within 24 hr postinfection; the majority of the suckling mouse (5/6) received with a dose of 2 × 10^7^ CFU died within 48 hr after. During the experimental observation period, some lactating mice injected with 2 × 10^6^ CFU also died (3/6), while those injected with 2 × 10^5^ CFU did not die. The LD_50_ of HBTS-1 in suckling mice was calculated as 3.1 × 10^6^ CFU using Karber's method. The control group was injected with PBS, and the adult mice all survived. These results clearly show that *E. miricola* HBTS-1 is pathogenic to suckling mice, suggesting its potential to infect other mammals.

### 3.5. The *E. miricola* Isolates Showed Severe Multidrug Resistance

The existence of multidrug resistance of *E. miricola* has already been documented in several literature [14, 18], which causes considerable trouble for the control and prevention of this pathogen. In order to clarify the prevalence of the antibiotic resistance of the bullfrog-derived *E. miricola* in Tangshan, we performed drug sensitivity tests for the 42 *E. miricola* isolates in this study. Although there is no standardized method to perform antimicrobial susceptibility testing for *E. miricola*, the data showed that the majority of the isolates were resistant to ampicillin, cefuroxime, ceftazidime, cefaclor, streptomycin, gentamicin, kanamycin, amikacin, tetracycline, ciprofloxacin, ofloxacin, nitrofurantoin, norfloxacin, and aztreonam without an apparent inhibition zone, as is shown in Figure 5(a). The data of antibiotic susceptibility testing of each isolate are illustrated in *Supplementary 2*. Taken together, the *E. miricola* isolates from Tangshan region displayed considerable antibiotic resistance, which brought challenges for antibiotic control of crooked head disease of bullfrogs in this region.

## 4. Discussion

*E. miricola*-caused crooked head disease was first reported in black-spotted frogs in 2016 in China [1]. Since then, this bacterium has been increasingly documented as an infectious pathogen in several other frog species with similar symptoms of neurological disorder, cataracts, and red legs [4]. Several cases of crooked head disease in bullfrogs were reported in 2023, and both *E. miricola* and *E. meningoseptica* were determined to be the causal pathogen [5, 14]. In 2023, serious outbreaks of meningitis-like disease occurred on bullfrog farms in Tangshan, a northern city of China. In some farms, this disease resulted in a total of 50% death of cultured bullfrogs. From the diseased frogs, we isolated strains of *E. miricola* and determined the etiological agents via the artificial infection experiment. Furthermore, *E. miricola* strains were isolated from the majority of sick frogs from different farms, suggesting that this bacterium is the major causative agent of crooked head disease in bullfrogs in Tangshan region.

Of note, this disease spread very fast, and almost half of the cultured bullfrogs got sick and died within 2 months after the onset, indicating its high infectivity. We collected swabs from the skin, oral cavity, and anus of sick frogs, and all the samples were determined *E. miricola* positive by PCR. Importantly, we also detected the existence of this bacterium in water from the habitat of sick frogs. A previous study had reported that immersion infection of *E. miricola* displayed high infection efficiency with 100% death in black-spotted frogs [1, 20]. Therefore, environmental disinfection, particularly water cleaning, may exert a pivotal role in the control of *E. miricola* caused-infection.

In this study, we successfully reproduced crooked head disease in bullfrogs by oral feeding, intramuscular injection, intraperitoneal injection, or immersion infection, suggesting multiple routes of exposure may be involved in this disease. Neurological disorders are usually associated with *E. miricola* infection in frogs, and the brain was reported as the main target organ of this pathogen [21]. However, *E. miricola* infection is not limited to brain tissue. In this study, we isolated *E. miricola* from the blood, brain, liver, spleen, eyes, and even muscles, supported by the results of histopathological analysis, suggesting a systematic septic infection occurred.

As a significant source of meat, bullfrogs are known to harbor a variety of pathogenic bacteria, such as *Aeromonas hydrophila*, *Vibrio cholerae*, *Streptococcus agalactiae*, *Edwardsiella tarda*, and *Elizabethkingia* spp. [22], which are also capable of causing infections in both mammals and humans, posing a potential threat to human health. Here, we noticed that, although the optimal culture temperature of *E. miricola* isolate HBTS-1 is 30°C, they grew well at 37°C, close to the body temperature of mammalian animals. Therefore, we attempted to infect KM mice with the HBST-1 isolate. Interestingly, the adult vaccinated mice showed no abnormalities, while 5-day-old suckling mice developed a red body and died within 72 hr post intramuscular injection, with *E. miricola* isolated from organs including the brain. The unexpected data suggest that the bullfrog-derived *E. miricola* isolate may have potential pathogenicity in mammals. Interestingly, although cases of *E. miricola* infections in humans are increasing, it occurs mainly in immunocompromised populations, such as elderly people, newborn, and children [23], and these reports have some similarities with our data.

*E. miricola* has the ability to cross the blood–brain barrier, and thus, mainly resulted in brain damage neurological disorders [24]. However, the most antibiotics are unable to cross the blood–brain barrier, brings great difficulty in the treatment of this bacterium. Moreover, *E. miricola* isolates either from human and frogs showed multiantibotic reisitance [19, 25], which aggravated the limitation of antibiotics in *E. miricola* treatment. *E. miricola* also showed resistance to a variety of antibiotics.

## 5. Conclusion

In this paper, we reported outbreaks of *E. miricola* caused neurologic disorders and cataracts in cultured bullfrogs in Tangshan city, Hebei Province. From diseased bullfrogs of 10 farms, we isolated 42 strains of *E. miricola*. The isolate could reproduce fatal meningitis-like disease in bullfrogs and displayed highly pathogenicity in both bullfrogs and suckling mice, indicating its potential threat to mammals. Antibiotic sensitization tests of the isolates were also performed in this study. These findings prioritized *E. miricola* as important zoonotic agents, which may have ramifications for human medicine.

## Figures and Tables

**Table 1:** Primers for detection of *E. miricola* virulence genes.

Virulence genes	Primer sequence (5′-3′)	Tm	Product size (bp)
*fabG*	ATGAAACTATTAGAAGGAAAAGTAG	51	744
	CTAAGTTAACATTCCGCCA		

*fabV*	ATGATCATACAACCACGTGTTA	53	1,203
	TTATCCTTCTATACTTGGGATGT		

*wecB*	ATGAAGAAACTAAAAGTAATGACG	53	1,140
	TTAAATTTCTTCAGACCAGACAG		

*ureB*	ATGATACCAGGAGAAATTTTTGT	54	369
	TTACAGGTTTTTAAAATTTAATTGA		

*aceA*	ATGAAAACTATTCAGGAACTACAAC	54	1,275
	TTAGAATTGTGCTGTTTCTGTAGA		

*acyl*	ATGTCAGACATCGCATCAA	51	240
	TTATTTGTTGACTACTTCTTCAAT		

*hly*	ATGGGCAGGACAGTTGTC	52	174
	TTATTTTCCTAGTTCTAATTGATT		

*wbpO*	ATGAACAATCACCGAATAGC	53	1,293
	TTATAATCTTGCATCTACGATCTC		

**Table 2:** Biochemical characteristics of isolated strain HBTS-1.

Items	Reactions	
	HBTS-1	*E. miricola* (CM160701) [2]
Gram stain	—	—
Cellular morphology	rod	rod
Motility	—	—
Hemolytic reaction	+	—
Oxidase	+	+
Catalase	+	+
Ornithine decarboxylase	+	nd
Simmons citrate	—	nd
Glucose	+	+
Maltose	+	+
Mannose	+	+
Lysine decarboxylase	+	nd
Sucrose	+	+
Lactose	+	+
Arginine dihydrolase	—	nd
Arabinose	—	—
Raffinose	—	—
Sorbitol	+	+
Phenylalanine	—	nd
Sorbitol	—	—
Urease	+	+
Inositol	+	nd
Adonitol	—	nd
Tryptophan deaminase	—	nd
Indole production	+	+

*Note*: +, positive; −, negative; v, variable reactions; nd, no data available.

## Data Availability

The original contributions presented in the study are included in the article/Supplementary Material. Further inquiries can be directed to the corresponding authors.

## Supplementary Materials

Supplementary 1 The information of geographical distribution and coordinates of bullfrog farms sampled.

Supplementary 2 The antibotic susceptibility test results of the 42 isolates.

## References

[1] Hu R., Yuan J., Meng Y., Wang Z., Gu Z. (2017). Pathogenic *Elizabethkingia miricola* infection in cultured black-spotted frogs, China, 2016. *Emerging Infectious Diseases*.

[2] Lei X. P., Yi G., Wang K. Y. (2019). *Elizabethkingia miricola* infection in Chinese spiny frog (*Quasipaa spinosa*). *Transboundary and Emerging Diseases*.

[3] Yang S., Si C., Mani R., Keller J., Hoenerhoff M. J. (2023). Septicemia caused by an emerging pathogen, *Elizabethkingia miricola*, in a laboratory colony of African dwarf frogs (*Hymenochirus curtipes*). *Veterinary Pathology*.

[4] Trimpert J., Eichhorn I., Vladimirova D. (2021). *Elizabethkingia miricola* infection in multiple anuran species. *Transboundary and Emerging Diseases*.

[5] Tsai M.-A., See M. S., Chiu C.-H., Wang P.-C., Chen S.-C. (2023). Genotypic and phenotypic analysis of *Elizabethkingia meningoseptica* in bullfrog *Rana catesbeiana* isolated in Taiwan. *Journal of Fish Diseases*.

[6] Lin J.-N., Lai C.-H., Yang C.-H., Huang Y.-H. (2019). *Elizabethkingia* infections in humans: from genomics to clinics. *Microorganisms*.

[7] Zdziarski P., Paściak M., Rogala K., Korzeniowska-Kowal A., Gamian A. (2017). *Elizabethkingia miricola* as an opportunistic oral pathogen associated with superinfectious complications in humoral immunodeficiency: a case report. *BMC Infectious Diseases*.

[8] Zhuo X., Zhou Y., Liu L. (2023). Acute bacterial encephalitis complicated with recurrent nasopharyngeal carcinoma associated with *Elizabethkingia miricola* infection: a case report. *Frontiers in Neurology*.

[9] Green O., Murray P., Gea-Banacloche J. C. (2008). Sepsis caused by *Elizabethkingia miricola* successfully treated with tigecycline and levofloxacin. *Diagnostic Microbiology and Infectious Disease*.

[10] Howard J. C., Chen K., Anderson T., Dalton S. C. (2020). *Elizabethkingia miricola* bacteraemia in a haemodialysis patient. *Access Microbiology*.

[11] Calatrava E., Casanovas I., Foronda C., Cobo F. (2020). Joint infection due to *Elizabethkingia miricola*. *Revista Española de Quimioterapia*.

[12] Badawi K., Deskins S., Catherman K., Lastinger A. (2022). Out of this world: *Elizabethkingia miricola* complicated urinary tract infection in a patient with associated pubic symphysis osteomyelitis and pyomyositis. *IDCases*.

[13] Weese J. S., Sobkowich K. E., Poljak Z., Bernardo T. M. (2023). Isolation of *Elizabethkingia* spp. from diagnostic specimens from dogs and cats, United States, 2019–2021. *Emerging Infectious Diseases*.

[14] Wei D., Cheng Y., Xiao S. (2023). Natural occurrences and characterization of *Elizabethkingia miricola* infection in cultured bullfrogs (*Rana catesbeiana*). *Frontiers in Cellular and Infection Microbiology*.

[15] Tamura K., Stecher G., Kumar S., Battistuzzi F. U. (2021). MEGA11: molecular evolutionary genetics analysis version 11. *Molecular Biology and Evolution*.

[16] Sneath P. H., Sokal R. R. (1962). Numerical taxonomy. *Nature*.

[17] Kimura M. (1980). A simple method for estimating evolutionary rates of base substitutions through comparative studies of nucleotide sequences. *Journal of Molecular Evolution*.

[18] Li S., Wang X., Lu Y. (2023). Co-infections of *Klebsiella pneumoniae* and *Elizabethkingia miricola* in black-spotted frogs (*Pelophylax nigromaculatus*). *Microbial Pathogenesis*.

[19] Burnard D., Gore L., Henderson A. (2020). Comparative genomics and antimicrobial resistance profiling of *Elizabethkingia* isolates reveal nosocomial transmission and in vitro susceptibility to fluoroquinolones, tetracyclines, and trimethoprim-sulfamethoxazole. *Journal of Clinical Microbiology*.

[20] Qiao M., Zhang L., Xu C., Huo X., Chang J., Su J. (2022). Chitosan and anisodamine enhance the immersion immune efficacy of inactivated *Elizabethkingia miricola* vaccine in black spotted frogs. *Fish & Shellfish Immunology*.

[21] Huang X., Feng Y., Tang H. (2019). Candidate animal disease model of *Elizabethkingia* spp. infection in humans, based on the systematic pathology and oxidative damage caused by *E. miricola* in *Pelophylax nigromaculatus*. *Oxidative Medicine and Cellular Longevity*.

[22] Zepeda-Velazquez A. P., Gómez-De-Anda F.-R., Aguilar-Mendoza L. F. (2023). Bullfrogs (*Lithobates catesbeianus*) as a potential source of foodborne disease. *Journal of Food Protection*.

[23] Dziuban E. J., Franks J. L., So M., Peacock G., Blaney D. D. (2018). Elizabethkingia in children: a comprehensive review of symptomatic cases reported from 1944 to 2017. *Clinical Infectious Diseases*.

[24] Li F., Chen B., Xu M. (2023). Immune activation and inflammatory response mediated by the NOD/toll-like receptor signaling pathway—the potential mechanism of bullfrog (*Lithobates catesbeiana*) meningitis caused by *Elizabethkingia miricola*. *International Journal of Molecular Sciences*.

[25] Liu F., Hou J., Yu F., Gu Z., Hu R. (2023). Identification and pathogenicity of multidrug-resistant *Elizabethkingia miricola* isolated from farmed American bullfrogs *Rana catesbeiana* in China with in vitro screening of herbal antimicrobial agents. *Journal of Aquatic Animal Health*.

