# Supplementary material for: Outbreaks of Elizabethkingia miricola Caused Fatal Meningitis-Like Disease in Cultured Bullfrogs
Source: Transbound Emerg Dis. 2024 Apr 22;2024:4733320. doi: 10.1155/2024/4733320 (PMC12016771; doi:10.1155/2024/4733320)
Supplement: Supplementary 1 — The information of geographical distribution and coordinates of bullfrog farms sampled. [file 4733320.f1.docx]

Table S1. The information of geographical distribution and coordinates of bullfrog farms sampled.

|  | Geographical location | Latitude and longitude | Number of isolated bacteria |
| --- | --- | --- | --- |
| Farm 1 | Liushuquan, Fengnan | 118.119477°E, 39.35477°N | 4 |
| Farm 2 | Bijiaquan, Fengnan | 118.093802°E, 39.272813°N | 4 |
| Farm 3 | Yuezhicun, Fengnan | 118.177985°E, 39.468908°N | 4 |
| Farm 4 | Huangtuo, Luannan | 118.414737°E, 39.731396°N | 5 |
| Farm 5 | Xiyutuo, Luannan | 118.44706°E, 39.356681°N | 4 |
| Farm 6 | Bogezhuang, Luannan | 118.539782°E, 39.306424°N | 4 |
| Farm 7 | Farm 4, Caofeidian | 118.405816°E, 39.236514°N | 4 |
| Farm 8 | Farm 7, Caofeidian | 118.351119°E, 39.217928°N | 3 |
| Farm 9 | Zengjiawan, Caofeidian | 118.419506°E, 39.382571°N | 4 |
| Farm 10 | Xianghecun, Caofeidian | 118.54187°E, 39.230479°N | 6 |
