# Supplementary material for: Outbreaks of Elizabethkingia miricola Caused Fatal Meningitis-Like Disease in Cultured Bullfrogs
Source: Transbound Emerg Dis. 2024 Apr 22;2024:4733320. doi: 10.1155/2024/4733320 (PMC12016771; doi:10.1155/2024/4733320)
Supplement: Supplementary 2 — The antibotic susceptibility test results of the 42 isolates. [file 4733320.f2.docx]

Table S2. The antibotic susceptibility test results of the 42 isolates.

|  |  | Inhibitory Diameter (mm) | | | | | | | | | | | | | | | | | | | | | |
| --- | --- | --- | --- | --- | --- | --- | --- | --- | --- | --- | --- | --- | --- | --- | --- | --- | --- | --- | --- | --- | --- | --- | --- |
| Strain name | Separating regions | AM | CTR | CAZ | CXM | CEC | AT | MEM | GM | AK | K | S | TE | D | MI | CIP | NOR | OFX | TMP | C | FD | RA | FON |
| HBTS-1 | Liushuquan, Fengnan | 6 | 6 | 6 | 6 | 6 | 6 | 10 | 6 | 6 | 6 | 9 | 6 | 9 | 15 | 6 | 9 | 10 | 10 | 13 | 6 | 20 | 20 |
| HBTS-2 | Liushuquan, Fengnan | 6 | 6 | 6 | 6 | 6 | 6 | 11 | 6 | 10 | 6 | 6 | 8 | 9 | 15 | 6 | 9 | 6 | 6 | 12 | 6 | 24 | 13 |
| HBTS-3 | Liushuquan, Fengnan | 6 | 6 | 6 | 6 | 6 | 6 | 10 | 6 | 6 | 6 | 6 | 10 | 10 | 15 | 6 | 6 | 6 | 10 | 14 | 6 | 25 | 19 |
| HBTS-4 | Liushuquan, Fengnan | 6 | 11 | 6 | 6 | 6 | 6 | 9 | 6 | 6 | 6 | 6 | 6 | 10 | 14 | 6 | 6 | 6 | 9 | 10 | 6 | 21 | 20 |
| HBTS-5 | Bijiaquan, Fengnan | 6 | 6 | 6 | 6 | 6 | 6 | 13 | 6 | 6 | 6 | 6 | 8 | 13 | 22 | 6 | 6 | 6 | 28 | 11 | 9 | 24 | 25 |
| HBTS-6 | Bijiaquan, Fengnan | 6 | 12 | 6 | 6 | 6 | 6 | 23 | 6 | 6 | 6 | 6 | 6 | 16 | 21 | 6 | 6 | 6 | 16 | 11 | 6 | 25 | 28 |
| HBTS-7 | Bijiaquan, Fengnan | 6 | 6 | 6 | 6 | 6 | 6 | 6 | 6 | 6 | 6 | 6 | 6 | 10 | 15 | 6 | 6 | 6 | 6 | 12 | 6 | 23 | 17 |
| HBTS-8 | Bijiaquan, Fengnan | 6 | 11 | 8 | 6 | 8 | 6 | 12 | 6 | 6 | 6 | 6 | 8 | 16 | 21 | 6 | 6 | 6 | 14 | 17 | 6 | 23 | 23 |
| HBTS-9 | Yuezhicun, Fengnan | 6 | 13 | 6 | 6 | 6 | 6 | 23 | 6 | 6 | 6 | 6 | 10 | 16 | 21 | 6 | 6 | 10 | 11 | 18 | 6 | 23 | 28 |
| HBTS-10 | Yuezhicun, Fengnan | 6 | 9 | 6 | 6 | 6 | 18 | 6 | 6 | 6 | 6 | 6 | 6 | 13 | 15 | 6 | 7 | 6 | 9 | 11 | 6 | 26 | 27 |
| HBTS-11 | Yuezhicun, Fengnan | 6 | 6 | 6 | 6 | 6 | 6 | 9 | 6 | 6 | 6 | 6 | 6 | 10 | 15 | 6 | 6 | 6 | 11 | 10 | 6 | 19 | 18 |
| HBTS-12 | Yuezhicun, Fengnan | 6 | 9 | 8 | 6 | 8 | 18 | 10 | 6 | 6 | 6 | 6 | 6 | 13 | 20 | 8 | 6 | 6 | 11 | 14 | 6 | 22 | 23 |
| HBTS-13 | Huangtuo, Luannan | 6 | 6 | 6 | 6 | 6 | 6 | 10 | 6 | 6 | 6 | 6 | 6 | 8 | 15 | 6 | 6 | 6 | 8 | 6 | 6 | 18 | 19 |
| HBTS-14 | Huangtuo, Luannan | 6 | 6 | 6 | 6 | 6 | 6 | 13 | 8 | 6 | 6 | 6 | 6 | 10 | 15 | 6 | 6 | 6 | 10 | 10 | 10 | 20 | 19 |
| HBTS-15 | Huangtuo, Luannan | 6 | 6 | 6 | 6 | 6 | 6 | 10 | 6 | 6 | 6 | 6 | 6 | 8 | 15 | 6 | 6 | 6 | 6 | 10 | 6 | 23 | 20 |
| HBTS-16 | Huangtuo, Luannan | 6 | 9 | 6 | 6 | 6 | 6 | 11 | 6 | 6 | 9 | 6 | 6 | 13 | 21 | 6 | 9 | 10 | 14 | 12 | 10 | 32 | 23 |
| HBTS-17 | Huangtuo, Luannan | 6 | 9 | 6 | 6 | 6 | 6 | 11 | 6 | 6 | 6 | 6 | 10 | 10 | 15 | 6 | 6 | 6 | 10 | 12 | 6 | 21 | 20 |
| HBTS-18 | Xiyutuo, Luannan | 6 | 21 | 6 | 6 | 6 | 6 | 23 | 6 | 6 | 6 | 6 | 6 | 10 | 14 | 6 | 6 | 7 | 18 | 14 | 6 | 21 | 21 |
| HBTS-19 | Xiyutuo, Luannan | 6 | 6 | 6 | 6 | 6 | 6 | 10 | 6 | 6 | 6 | 6 | 6 | 10 | 15 | 6 | 10 | 6 | 8 | 10 | 6 | 20 | 20 |
| HBTS-20 | Xiyutuo, Luannan | 6 | 6 | 6 | 6 | 6 | 6 | 21 | 6 | 10 | 6 | 6 | 6 | 13 | 14 | 6 | 6 | 6 | 10 | 10 | 6 | 20 | 17 |
| HBTS-21 | Xiyutuo, Luannan | 6 | 6 | 6 | 6 | 6 | 6 | 10 | 6 | 9 | 6 | 6 | 6 | 10 | 15 | 6 | 6 | 6 | 10 | 9 | 6 | 20 | 20 |
| HBTS-22 | Bogezhuang, Luannan | 6 | 22 | 8 | 6 | 8 | 6 | 11 | 6 | 6 | 6 | 6 | 6 | 10 | 15 | 6 | 6 | 6 | 20 | 9 | 6 | 28 | 11 |
| HBTS-23 | Bogezhuang, Luannan | 6 | 6 | 6 | 6 | 6 | 6 | 9 | 6 | 6 | 6 | 6 | 9 | 9 | 12 | 11 | 6 | 9 | 9 | 17 | 6 | 18 | 20 |
| HBTS-24 | Bogezhuang, Luannan | 10 | 21 | 6 | 6 | 6 | 6 | 23 | 6 | 6 | 9 | 10 | 9 | 20 | 25 | 6 | 6 | 9 | 10 | 16 | 6 | 18 | 23 |
| HBTS-25 | Bogezhuang, Luannan | 6 | 10 | 8 | 10 | 9 | 6 | 10 | 10 | 6 | 6 | 6 | 10 | 13 | 20 | 6 | 10 | 10 | 13 | 19 | 6 | 27 | 23 |
| HBTS-26 | Farm 4, Caofeidian | 6 | 11 | 6 | 6 | 6 | 6 | 9 | 6 | 6 | 6 | 6 | 6 | 10 | 20 | 6 | 6 | 6 | 10 | 11 | 6 | 19 | 20 |
| HBTS-27 | Farm 4, Caofeidian | 6 | 11 | 6 | 6 | 8 | 6 | 13 | 6 | 9 | 6 | 6 | 8 | 13 | 19 | 6 | 6 | 6 | 10 | 10 | 6 | 20 | 24 |
| HBTS-28 | Farm 4, Caofeidian | 6 | 6 | 6 | 6 | 6 | 6 | 13 | 6 | 6 | 6 | 10 | 10 | 10 | 11 | 6 | 22 | 6 | 6 | 14 | 6 | 1 | 13 |
| HBTS-29 | Farm 4, Caofeidian | 6 | 9 | 6 | 6 | 6 | 6 | 11 | 6 | 6 | 6 | 6 | 10 | 13 | 15 | 6 | 6 | 6 | 8 | 12 | 10 | 21 | 20 |
| HBTS-30 | Farm 7, Caofeidian | 6 | 6 | 6 | 6 | 6 | 6 | 13 | 6 | 6 | 6 | 6 | 6 | 9 | 14 | 6 | 9 | 6 | 10 | 13 | 6 | 20 | 20 |
| HBTS-31 | Farm 7, Caofeidian | 6 | 10 | 6 | 6 | 6 | 6 | 9 | 6 | 6 | 6 | 6 | 9 | 13 | 14 | 6 | 6 | 6 | 10 | 15 | 6 | 22 | 20 |
| HBTS-32 | Farm 7, Caofeidian | 6 | 10 | 6 | 6 | 9 | 6 | 11 | 6 | 6 | 6 | 6 | 9 | 13 | 20 | 6 | 23 | 6 | 18 | 10 | 10 | 25 | 25 |
| HBTS-33 | Zengjiawan, Caofeidian | 6 | 9 | 6 | 6 | 6 | 6 | 10 | 6 | 6 | 6 | 6 | 9 | 13 | 18 | 6 | 6 | 8 | 10 | 14 | 6 | 22 | 23 |
| HBTS-34 | Zengjiawan, Caofeidian | 6 | 9 | 6 | 6 | 6 | 6 | 21 | 6 | 6 | 6 | 6 | 10 | 13 | 14 | 6 | 6 | 6 | 15 | 14 | 6 | 20 | 25 |
| HBTS-35 | Zengjiawan, Caofeidian | 6 | 6 | 6 | 6 | 6 | 6 | 11 | 6 | 6 | 6 | 6 | 6 | 10 | 13 | 6 | 6 | 6 | 13 | 10 | 6 | 19 | 25 |
| HBTS-36 | Zengjiawan, Caofeidian | 6 | 6 | 6 | 6 | 6 | 6 | 7 | 6 | 6 | 6 | 6 | 6 | 10 | 11 | 6 | 7 | 6 | 8 | 6 | 6 | 10 | 20 |
| HBTS-37 | Xianghecun, Caofeidian | 6 | 6 | 6 | 6 | 6 | 6 | 22 | 6 | 6 | 6 | 6 | 10 | 10 | 14 | 6 | 6 | 6 | 15 | 18 | 6 | 17 | 25 |
| HBTS-38 | Xianghecun, Caofeidian | 6 | 10 | 6 | 6 | 6 | 19 | 10 | 6 | 9 | 6 | 6 | 6 | 17 | 17 | 6 | 6 | 6 | 10 | 10 | 6 | 23 | 21 |
| HBTS-39 | Xianghecun, Caofeidian | 6 | 6 | 6 | 6 | 6 | 6 | 10 | 6 | 6 | 6 | 6 | 10 | 9 | 11 | 6 | 6 | 7 | 10 | 13 | 6 | 20 | 18 |
| HBTS-40 | Xianghecun, Caofeidian | 6 | 6 | 6 | 6 | 6 | 6 | 22 | 6 | 6 | 6 | 6 | 6 | 9 | 11 | 6 | 6 | 6 | 8 | 10 | 6 | 22 | 20 |
| HBTS-41 | Xianghecun, Caofeidian | 6 | 21 | 6 | 6 | 6 | 6 | 8 | 6 | 6 | 6 | 6 | 7 | 7 | 10 | 6 | 6 | 6 | 14 | 13 | 6 | 24 | 11 |
| HBTS-42 | Xianghecun, Caofeidian | 6 | 6 | 6 | 6 | 6 | 6 | 6 | 6 | 6 | 6 | 6 | 6 | 13 | 18 | 6 | 6 | 6 | 15 | 10 | 6 | 23 | 12 |

Note: Ampicillin (AM), Ceftriaxone (CTR), Ceftazidime (CAZ), Cefuroxime (CXM), Cefaclor (CEC), Aztreonam (AT), Meropenem (MEM), Gentamicin (GM), Amikacin (AK), Kanamycin (K), Streptomycin (S), Tetracycline (TE), Doxycycline (D), Minocycline (MI), Ciprofloxacin (CIP), Norfloxacin (NOR), Ofloxacin (OFX), Trimethoprim (TMP), Chloramphenicol (C), Nitrofurantoin (FD), Rifampicin (RA), Florfenicol (FON).
